# A shikonin-based hydrogel dressing exhibiting synergistic anti-inflammatory, anti-angiogenic, and anti-fibrotic effects for the prevention of hypertrophic scars

**DOI:** 10.3389/fimmu.2025.1736748

**Published:** 2026-01-14

**Authors:** Ziyu Wang, Yujie Zhao, Zhoujiang Qu, Hui Wang, Yuling Zhang, Yufang Liu, Chengde Li, Kun Wang, Guoqi Cao

**Affiliations:** 1 Department of Burns and Wound Repair, The First Affiliated Hospital of Shandong Second Medical University, Weifang People’s Hospital, Weifang, Shandong, China; 2 School of Pharmacy, Shandong Second Medical University, Weifang, Shandong, China; 3 School of Clinical Medicine, Shandong Second Medical University, Weifang, Shandong, China; 4 Department of Rheumatism and Immunity, The First Affiliated Hospital of Shandong Second Medical University, Weifang People’s Hospital, Weifang, Shandong, China

**Keywords:** anti-angiogenic, anti-fibrotic, anti-inflammatory, GelMA, hypertrophic scars, shikonin

## Abstract

**Introduction:**

The management and prevention of hypertrophic scars present significant challenges in clinical medicine. The development of hypertrophic scars primarily results from angiogenesis, localized inflammatory responses, and the excessive deposition of collagen during the wound healing process. Given the practical requirements for clinical applications, the development of a multifunctional dressing that exhibits synergistic effects and adapts to wounds of diverse shapes is of paramount importance. Hydrogels have emerged as promising biomaterials in dermatological applications due to their excellent biocompatibility, injectability, and strong adhesion. Shikonin (SHI), a compound derived from traditional Chinese medicine, exhibits anti-inflammatory and antioxidant properties that facilitate wound healing. It is posited that SHI possesses diverse pharmacological potential for the prevention and treatment of scars.

**Methods:**

Based on these characteristics, this study developed a methacrylated gelatin (GelMA) dressing incorporated with shikonin. In vitro experiments were conducted to evaluate the material properties and biocompatibility of SHI-GelMA. Concurrently, an in vivo rabbit ear model was established to assess the efficacy of SHI-GelMA in inhibiting scar hyperplasia through treatment and subsequent observation.

**Results:**

By utilizing GelMA’s superior biological properties, the dressing enhances the therapeutic efficacy of SHI on hypertrophic scars while addressing challenges related to drug release kinetics. In vitro experiments demonstrated that the SHI-GelMA dressing exhibits favorable biocompatibility. Importantly, both in vitro and in vivo studies consistently indicated that SHI-GelMA synergistically prevents and inhibits hypertrophic scar formation through SHI's principal anti-inflammatory, anti-angiogenic, and anti-fibrotic mechanisms.

**Discussion:**

In summary, we have successfully developed a functional wound dressing that incorporates the diverse bioactivities of SHI, offering novel materials and strategies for the synergistic prevention and treatment of hypertrophic scars.

## 1 Introduction

Skin tissue damage is a prevalent pathological condition encountered in clinical diagnosis and treatment, and an abnormal healing process often results in the formation of pathological scars (1–3). Hypertrophic scars (HS) serve as a typical example of fibrotic diseases, with epidemiological data indicating that the overall incidence of HS ranges from 4% to 16%, while the incidence among postoperative burn patients can be as high as 70% (4). The characteristics of HS include persistent inflammatory responses and abnormal deposition of extracellular matrix (ECM) (5–9). The process of skin wound healing can be divided into four dynamic stages: hemostasis, inflammation, proliferation, and remodeling (10). In normal physiological healing, fibroblasts play a crucial role under the regulation of cytokines such as Transforming Growth Factor Beta (TGF-β), secreting collagen type I (COL I), collagen type III (COL III), and α-smooth muscle actin (α-SMA) to maintain structural balance within the ECM (11–14). However, during the formation of HS, the sustained activation of pathological fibroblasts leads to an imbalance between collagen synthesis and degradation, and their excessive contractile properties further contribute to the phenomenon of scar contraction (15, 16). HS not only impairs physiological functions, such as the integrity of the skin barrier and joint mobility. But its significant deformities also adversely affect the psychological well-being of patients, resulting in dual suffering both physically and mentally (17).

Currently, several treatment methods are employed in clinical practice. Among these, corticosteroid injections and anti-proliferative drug injections are often associated with considerable pain during administration and are prone to causing pigmentation changes (18, 19). Laser treatment, while effective, is costly and may provoke erythema or blisters (20, 21). Radiation therapy poses a potential risk of carcinogenesis, and its long-term safety has not been thoroughly evaluated (22). Surgical interventions have a high recurrence rate and may lead to more severe scarring (23). Therefore, the development of effective early intervention strategies is of great clinical significance. For these reasons, we believe it is essential to design an optimal wound dressing that can effectively prevent the onset of HS in the early stages of wound formation.

A variety of biomaterials have been investigated for use in skin dressings, with hydrogels currently at the forefront of research (24–27). An ideal hydrogel scaffold for preventing HS should possess several key characteristics: excellent biocompatibility, injectability, and adhesiveness. These attributes enhance the clinical applicability of hydrogels in wound adhesion. Concurrently, the physical barrier created by the hydrogel effectively isolates the wound and prevents bacterial infection. Infection constitutes a critical factor that induces and exacerbates persistent inflammation, with the sustained inflammatory response serving as the primary initiating signal that drives fibroblast overactivation and subsequent scar formation. Furthermore, the porous structure of the hydrogel matrix allows for the absorption of a substantial amount of exudate, thereby providing a sustained moisturizing effect. Hydrogels demonstrate significant hydrophilicity, a high drug loading capacity, and sustained drug release characteristics, which contribute to prolonging the efficacy of the treatment area. Despite the numerous advantages of hydrogels as wound dressing materials, their effectiveness in the wound healing process is significantly influenced by the therapeutic agents incorporated within them (28).

Shikonin (SHI) is the primary bioactive compound extracted from the roots of Lithospermum erythrorhizon, commonly known as “Zicao”. As a traditional Chinese medicinal herb, it has been used for over two millennia to treat infections, inflammation, and hemorrhagic conditions (29–31). According to the principles of Traditional Chinese Medicine, SHI is believed to possess properties that cool the blood, promote circulation, and alleviate internal heat or fever. Recent scientific studies have validated its therapeutic applications (32). Contemporary research has shown that SHI’s chemical constituents are complex, exhibiting anticancer, anti-inflammatory, and wound-healing properties (33, 34). Previous studies have demonstrated that SHI can promote autophagy in myofibroblasts derived from HS and inhibit the occurrence and progression of scars by downregulating the expression of COL I, COL III, and α-SMA (35, 36). However, current research on SHI in scar treatment remains limited to its application as a standalone drug, and no studies have investigated the potential of using SHI to develop novel dressings.

In summary, this study aims to develop a functional dressing for the early prevention of HS. SHI is combined with widely used gelatin methacryloyl (GelMA) at an optimal concentration to create a new biomaterial, SHI-GelMA, which can deliver precise and consistent therapeutic effects. In the initial phase of the research, the biocompatibility, anti-angiogenic, anti-inflammatory and collagen deposition-inhibiting properties of SHI-GelMA were evaluated. Subsequently, SHI-GelMA was applied in a rabbits’ ear model for *in vivo* assessment. The primary innovation of this study resides in the development of a multidimensional synergistic therapeutic system. At the clinical application, this research represents the first instance of integrating SHI with GelMA hydrogel for the preventive intervention of HS, thereby addressing a critical gap in sequential treatment strategies. From a treatment strategy perspective, the study introduces the novel use of a biomimetic microenvironment, constructed by GelMA, to facilitate orderly tissue repair during the early healing phase. Concurrently, the sustained-release properties of GelMA enable SHI to precisely modulate the healing process during later stages. Additionally, this approach not only harnesses the multi-target pharmacological activities of SHI but also highlights the active synergistic role of the GelMA microenvironment in promoting moist healing and mechanical regulation. Ultimately, the study establishes a translational design framework that integrates material properties with pathological mechanisms. This application strategically aligns the hydrogel’s diverse characteristics with the key pathophysiological features of scar formation, offering an innovative, feasible, and clinically promising systematic solution for the proactive prevention of HS.

## 2 Materials and methods

### 2.1 SHI-GelMA preparation

2% (w/v) SHI solution was prepared by dissolving SHI in 75% ethanol. SHI is completely dissolved and has not formed suspended particles. Subsequently, 0.5 g, 0.75 g, and 1 g of foamy GelMA were individually weighed and dissolved in 10 mL of phosphate-buffered saline (PBS) containing SHI at concentrations of 0.1 mg/mL and 0.5 mg/mL, respectively. The centrifuge tubes containing the samples were incubated in a 50 °C water bath to promote dissolution. Thereafter, 0.02 g of the photoinitiator lithium phenyl-2,4,6-trimethylbenzoylphosphinate was added and thoroughly mixed using ultrasound. Finally, the prepared GelMA hydrogel and SHI-GelMA hydrogel solutions were sterilized by filtration through a 0.22 μm membrane and stored in the dark for subsequent use.

### 2.2 Morphological characterization

After preparing GelMA hydrogels at various concentrations, they were exposed to ultraviolet (UV) light (wavelength: 365 nm, power: 20 mW/cm²) for 5 seconds. The characteristics of the 5%, 7.5%, and 10% GelMA hydrogels were documented using a single-lens reflex camera (Nikon, Tokyo, Japan). Subsequently, both GelMA and SHI-GelMA hydrogels were freeze-dried, and their pore size, porosity, and SHI distribution were assessed using scanning electron microscopy (SEM, Philips XL-30, Amsterdam, Netherlands) at an acceleration voltage of 15 kV.

### 2.3 *In vitro* SHI release study

Consistent with established protocols, precisely 2.0 g of SHI-GelMA hydrogel was loaded into a pre-equilibrated dialysis membrane (MWCO 3.5 kDa) containing 5.0 mL. This assembly was then submerged in 10 mL release medium consisting of PBS containing 0.5% (w/v) Tween 80 surfactant. Aliquots were systematically withdrawn from the reservoir at defined timepoints for analytical quantification, with immediate replenishment of equal volumes to preserve hydrostatic equilibrium throughout the experimental duration. The release of SHI from the SHI-GelMA hydrogel was quantitatively analyzed using a UV-Vis spectrophotometer at a wavelength of 425 nm. The cumulative release amount of SHI was calculated using the following equation.


$$\text{SHI release ratio}(\%)\;=\frac{10\times {\text{C}}_{\text{n}}+0.5\times \sum_{\text{x}=1}^{\text{n}-1}{\text{C}}_{\text{x}}}{\text{Mass of SHI in hydrogel}}\times 100$$

where Cn represents the concentration of the released SHI (μg/mL) at time “n”.

### 2.4 Degradation assay

This study conducted a degradation kinetics analysis using SHI-GelMA hydrogels containing 0.5 mg/mL of SHI. SHI-GelMA hydrogels with concentration gradients of 5%, 7.5%, and 10% were immersed in PBS at a constant temperature of 37 °C to simulate physiological conditions. At predetermined time points, the residual dry mass was quantitatively measured using a freeze-drying weighing method.The degradation ratios were calculated using the following formula:


Degradation ratio(%)=the hydrogels' current weightinitial weight×100%

### 2.5 Mechanical assay

The compressive mechanical characteristics of photocrosslinked GelMA hydrogel specimens (Φ 10 × 3 mm) were quantitatively analyzed using a mechanical testing machine (Instron-5969, Instron, Canton, MA, United States). Unconfined compression tests were conducted under ambient conditions at a controlled displacement rate of 0.5 mm/min until specimen failure occurred, as indicated by the inflection point on the stress-strain profiles. The elastic modulus was derived from the linear elastic region (0-10% strain) of the stress-strain curves through linear regression analysis.

### 2.6 Viscosity assay

The tests were performed using a HAAKE MARS Rotational Rheometer with parallel-plate geometry (P20 TiL, 20 mm diameter) at 25°C. The GelMA hydrogel samples were examined for viscosity change over time at a shear rate of 10/s. The viscosity value was recorded every 10 s for 60s.

### 2.7 Biocompatibility analysis

The cytocompatibility evaluation of SHI-GelMA composite hydrogels was conducted using murine fibroblast cell line L929 (Cell Bank, Chinese Academy of Sciences). Weigh 100 mg of each sample, including the photo-crosslinked GelMA hydrogel, SHI-GelMA with an SHI concentration of 0.1 mg/mL and 0.5 mg/mL. Subsequently, immerse each sample in 10 mL of DMEM(Dulbecco’s Modified Eagle Medium) for 72 hours to prepare the extraction solutions. Cellular proliferation analysis was performed by seeding 2×10^4^ cells/cm² in 6-well culture plates with extract-conditioned media, maintained at 37 °C in 5% CO_2_ humidified atmosphere. After 72-hour incubation, cell viability was assessed through dual-fluorescence live/dead staining (Invitrogen, United States) with confocal laser scanning microscopy (Nikon). Quantitative cell viability metrics were obtained via automated image processing using ImageJ software. L929 cells were seeded at a density of 2.0×10^4^ cells/mL in 96-well plates containing 200 µL of either the respective extract solutions or regular DMEM medium. The cells were cultured for 24, 72, and 120 h. Cell viability was assessed using the CCK-8 assay (Dojindo, Kumamoto, Japan) according to the manufacturer’s protocol.

To observe the L929 cell morphology, samples after 3 days *in vitro* culture were washed thrice in PBS and the samples were fixed in 4% paraformaldehyde at room temperature for 30 min. Then, the cells were permeabilized with 0.2% (v/v) Triton X-100 in PBS for 10 min and stained with Alexa Fluor 546 phalloidin (1:40 dilution in PBS, Invitrogen) for 30 min in the dark, followed by staining of the cell nuclei with 0.8 mg/mL of 4′,6-diamidino-2-phenylindole (DAPI, Invitrogen) for 10 min in the dark. Thereafter, observations of cell spreading were conducted using the laser-scanning confocal microscopy (STELLARIS, Leica, German).

### 2.8 *In vitro* impact of SHI-GelMA hydrogel on fibroblast functional expression

The quantitative reverse transcription polymerase chain reaction (qRT-PCR) test was conducted to investigate the effects of SHI-GelMA hydrogel on fibroblast function *in vitro*. L929 cells were cultured using the extract solutions from 7.5% GelMA hydrogel or SHI-GelMA hydrogel (containing 0.5 mg/ml SHI) for 72 h and the gene expression levels of COL I, α-SMA and VEGF in the cells were assessed. Following the manufacturer’s instructions, total RNA was extracted using Trizol reagent (Invitrogen) according to the manufacturer’s instructions, and DNase treatment was performed to eliminate any potential DNA contamination. The RNA concentration was determined by measuring the absorbance at 260 nm, and complementary DNA was synthesized using a reverse transcription kit (Invitrogen). The PCR primers employed in this study are listed in **Table 1**. GAPDH (glyceraldehyde-3-phosphate dehydrogenase) was used as an internal control.

**Table 1:** Primers used in the qRT–PCR.

Gene	5’—3’	Primer
COL 1	Forward	CGATGGATTCCCGTTCGAGT
	Reverse	TTCGATGACTGTCTTGCCCC
COL III	Forward	CCACGGAAACACTGGTGGAC
	Reverse	GCCAGCTGCACATCAAGGAC
α-SMA	Forward	TGCTGACAGAGGCACCACTGAA
	Reverse	CAGTTGTACGTCCAGAGGCATAG
VEGF	Forward	AGGCTGACCCACGACAGAA
	Reverse	CTTTGGTCTGCATTCACATC

### 2.9 *In vitro* anti-inflammatory properties of SHI-GelMA hydrogel

5×10^4^ L929 cells were cultured in six-well plates using the extract solutions of 2mL from GelMA hydrogel or SHI-GelMA hydrogel (containing 0.5 mg/ml SHI) for 72 h. Subsequently, the cells were washed twice with PBS. Prior to treatment, the L929 cells were cultured using serum-free DMEM for 1 day. The L929 cells were exposed to 1 mg/mL lipopolysaccharide (LPS, Sigma-Aldrich, USA) for 4 h, after which samples were prepared over a 30-min period. The cells were harvested to assess the Tumor Necrosis Factor-alpha (TNF-α) and Interleukin-6 (IL-6) gene expression levels by qRT-PCR testing.

### 2.10 Rabbit ear scar model establishment and treatment

A total of twelve male New Zealand rabbits aged 8 to 10 weeks were used in this study and purchased from Qingdao Kangda Aibo Biotechnology Co., Ltd. (Qingdao, China). The animals were housed under controlled conditions (24 ± 2 °C, 50 ± 10% humidity, 12h light/dark cycle) and acclimatized for one week. All experiments and procedures were approved by Animal Ethics Committee of Shandong Second Medical University in China, and the approval number is 2025SDL579.

Wounds are arranged in two columns and three rows, extending from the tip to the base of the rabbit ear. In each row, the two parallel wounds constitute a single group. Consequently, each rabbit ear comprises the following three groups. The experimental rabbits were anesthetized using inhalation of 2% to 2.5% isoflurane. Once adequate anesthesia was confirmed, six circular defects, each with a diameter of 8 mm, were created on the inner surface of the ventral side of each ear, removing the dermis, epidermis, and perichondrium.

The first group received SHI-GelMA treatment (SHI-GelMA group), the second group received just GelMA treatment (GelMA group), and the third group without any treatment (blank group). Then hydrogels were gelated by UV light irradiation (365 nm, 20 mW/cm2) for 5 s. The surface of the skin was imaged at 0, 7, 14, and 28 days to evaluate the rates of the incidence of HS.

After 7 day observation period, four experimental rabbits were anesthetized using 2% to 2.5% isoflurane. Subsequently, sterile ophthalmic scissors were employed to incise the muscle layer, allowing for the excision of the wound and the surrounding tissue within a 3 mm margin. The fresh wound tissue was immediately fixed in 4% formaldehyde for subsequent dehydration and paraffin block preparation. At this point, the four rabbits collectively provided a total of 48 samples, resulting in an average of 16 samples per group. After 14 and 28 days, the same procedure was conducted on the remaining eight rabbits.

### 2.11 Scar Elevation Index

The Scar Elevation Index (SEI) is utilized to evaluate the condition of scars and the process of wound healing. This index is determined by measuring the distance from the highest point of the vertical scar tissue (a) to the surface of the surrounding normal skin (b) and to the surface of the ear cartilage. The specific methodology involves using ImageJ software for measurement, where SEI is calculated as SEI = a/b. In this formula, ‘a’ represents the vertical distance from the highest point of the scar tissue to the surface of the ear cartilage, while ‘b’ denotes the vertical distance from the surface of the surrounding normal skin to the surface of the ear cartilage. The assessment of the SEI is performed prior to injection and again at 7, 14 and 28 days post-injection.

### 2.12 Histological and immunofluorescence staining analyses

On days 7, 14 and 28, rabbits were euthanized and photographed. Skin samples were collected from the wound sites, which were fixed in 4% paraformaldehyde and embedded in paraffin for sectioning into 5-µm thick slices. Hematoxylin and eosin (HE) staining and Masson staining were conducted to assess histological changes and collagen deposition.

During the immunohistochemical analysis, the samples were initially treated with 3% hydrogen peroxide for 10 minutes, followed by blocking with 5% bovine serum albumin at room temperature for 30 minutes. Subsequently, the samples were incubated overnight at 4 °C with mouse anti-rabbit monoclonal antibodies specific to COL I and COL III. Thereafter, the samples were incubated with secondary antibodies at room temperature for 1 hour. Finally, the tissue sections were developed using a DAB chromogenic reagent. Additionally, immunofluorescence staining was performed to assess the expression levels of α-SMA and vascular endothelial growth factor (VEGF).

### 2.13 *In vivo* impact of SHI-GelMA hydrogel

This study employed quantitative reverse transcription qRT-PCR to investigate the effects of SHI-GelMA hydrogel on samples *in vivo*. The gene expression levels of COL I, COL III, α-SMA, and VEGF by 28 days were assessed.

### 2.14 Statistical analysis

All experimental procedures were repeated at least three times. Values are presented as the mean ± standard deviation. Statistical significance was evaluated by one-way analysis of variance with GraphPad Prism software. A p value of <0.05 was deemed statistically significant.

## 3 Results

### 3.1 SHI-GelMA hydrogel characterization

**Figure 1:**
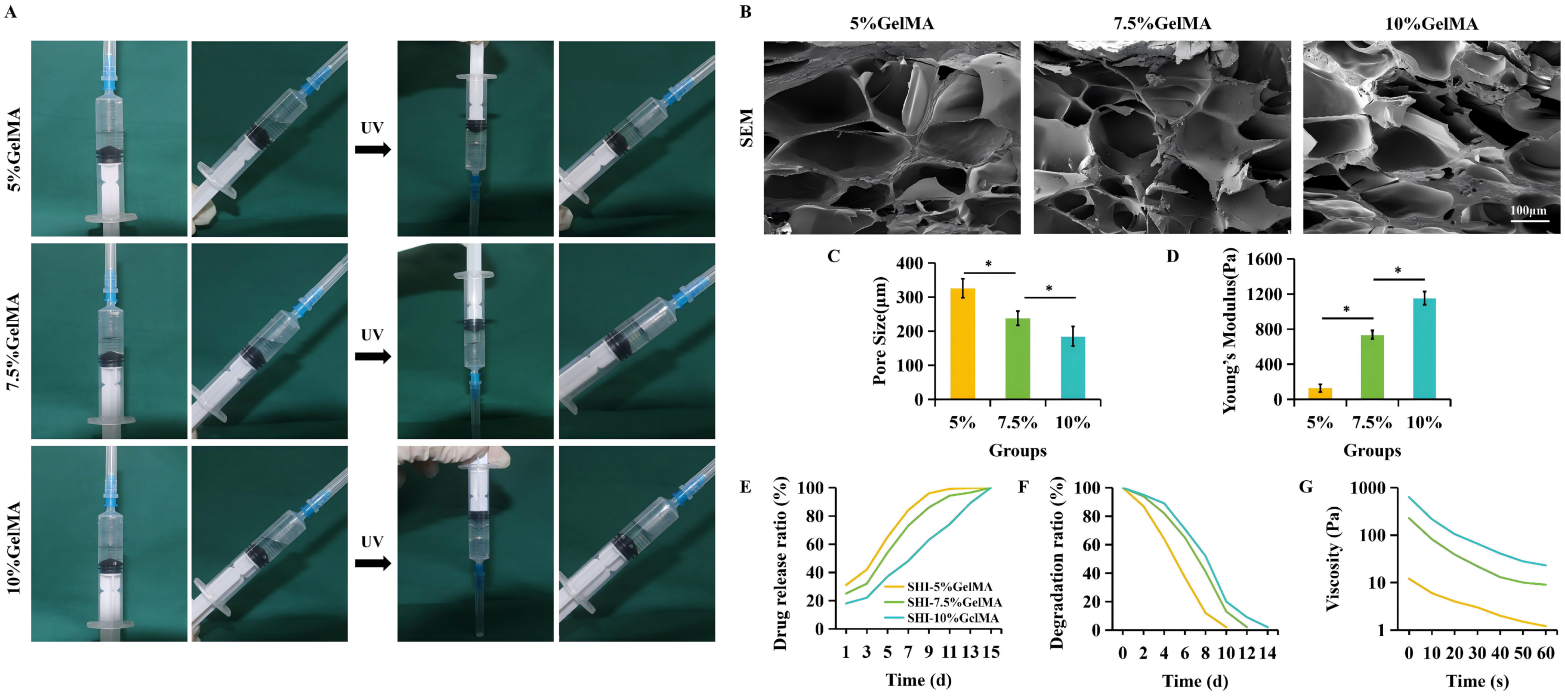
SHI-GelMA hydrogel characterization. **(A)** Appearance of GelMA at different concentrations before and after UV raying. **(B)** SEM images of different GelMA concentrations. Scale bar = 100μm. **(C)** Mean pore size of different GelMA concentrations. **(D)** Young’s modulus of different GelMA concentrations. **(E)** Drug release ratio of different GelMA concentrations with time. **(F)** Degration ratio of different GelMA concentrations with time. **(G)** Viscosity of different GelMA concentrations. (*P<0.05).

To develop more effective wound dressings, we prepared GelMA hydrogels at concentrations of 5%, 7.5%, and 10%. Following UV irradiation, all three groups of hydrogels demonstrated favorable gelation characteristics. SEM images revealed that the freeze-dried GelMA hydrogels across all three concentrations exhibited a highly interconnected and continuous microporous structure; however, the pore size distribution in the 10% GelMA hydrogels was inhomogeneous. The average pore sizes for the 5%, 7.5%, and 10% GelMA hydrogels were 326.2 μm, 238.6 μm, and 185.7 μm, respectively, indicating a gradual decrease in pore size with increasing concentration (**Figures 1A–C**).

As the concentration of GelMA increases, the release rate of SHI exhibits a decreasing trend. After a period of 14 days, nearly all SHI in the 10% GelMA group is released, a finding that aligns with the degradation rate of the GelMA hydrogel. This observation suggests a strong correlation between the degradation process of the GelMA hydrogel and the release of SHI, thereby facilitating the sustained therapeutic effects of SHI throughout the process (**Figures 1E, F**). The viscosity of the 7.5% and 10% GelMA groups was higher than that of the 5% GelMA group (**Figure 1G**). Additionally, the increase in GelMA concentration significantly enhances the elastic modulus of the material (**Figure 1D**).

Following an extensive assessment of various factors including morphology, SHI release efficiency, degradation rate, mechanical properties, and rheological characteristics, we have determined that a GelMA concentration of 7.5% is optimal for subsequent investigations. This concentration exhibited superior performance in terms of viscosity and pore distribution.

### 3.2 SHI-GelMA hydrogel biocompatibility

**Figure 2:**
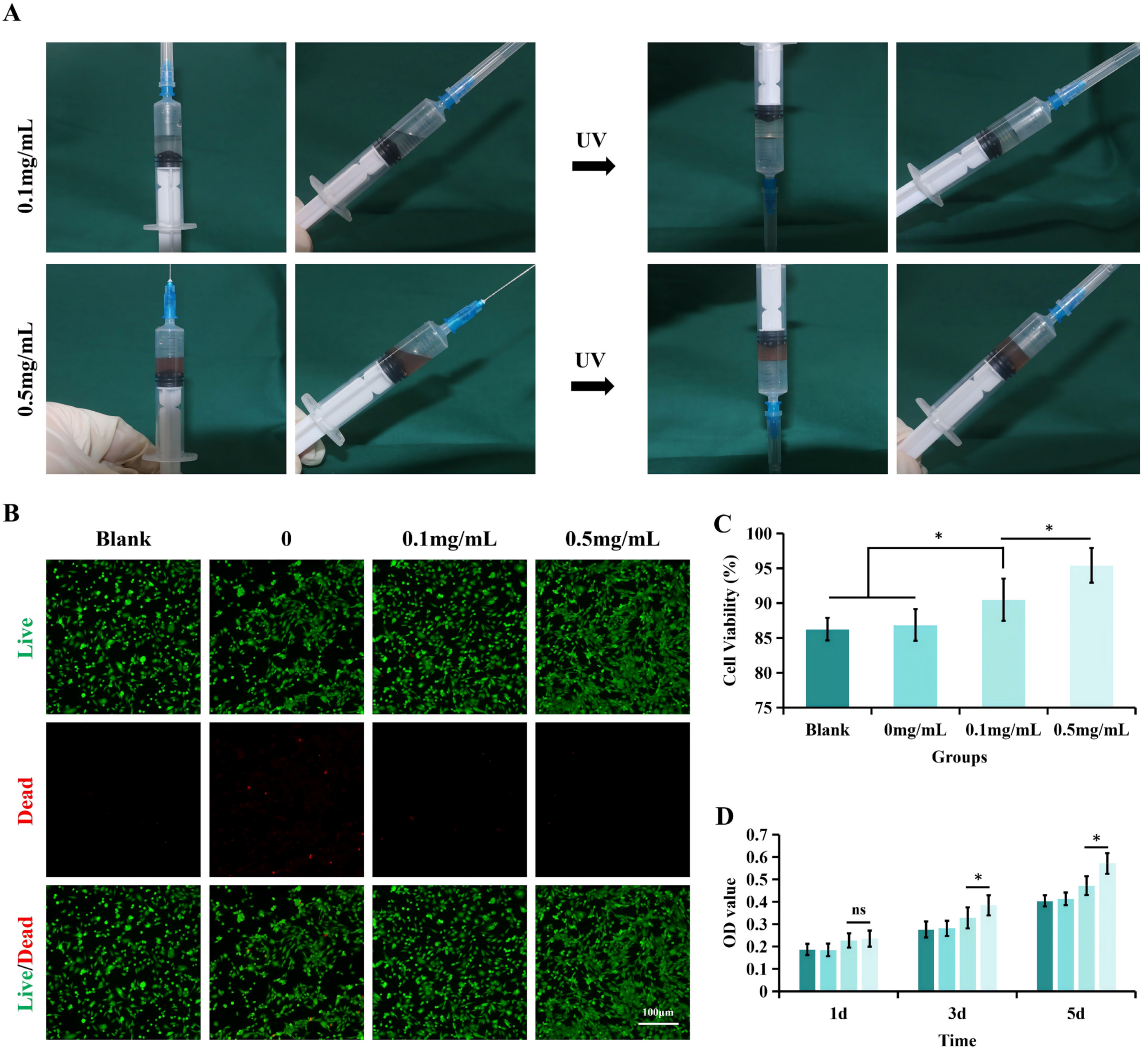
SHI-GelMA hydrogel biocompatibility. **(A)** Fluorescence images of Live/Dead cell staining and **(B)** quantification of cell survival area after L929 treated with different SHI concentrations at 3 days. Scale bar = 100μm. **(C)** Cell proliferation assay via CCK-8 test after L929 treated with different SHI concentrations at 1, 3, and 5 days. (*P<0.05, ns=no statistical difference)

**Figure 3:**
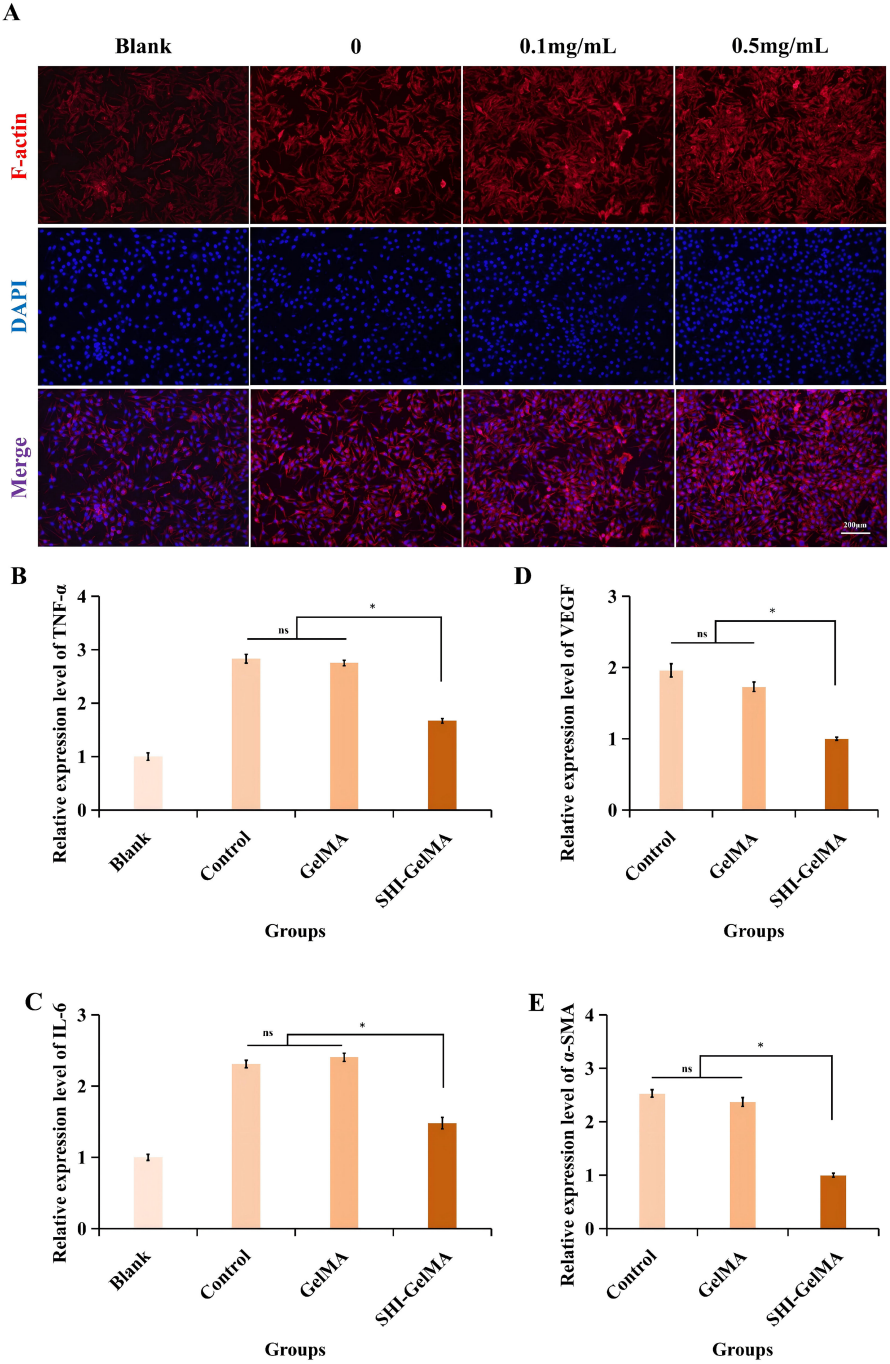
Biological function of SHI-GelMA hydrogel and qRT-PCR analysis of L929 cells. **(A)** Spreading of L929 cells. The expression levels of VEGF **(B)**, α-SMA **(C)**, TNF-α **(D)** and IL-6 **(E)** gene. (*P<0.05, ns, no statistical difference).

This investigation systematically examined the impact of SHI-GelMA hydrogels and varying concentrations of SHI on cell viability and proliferation, with the objective of evaluating the safety profile of SHI-GelMA hydrogels. Fluorescent imaging techniques for live/dead cell staining, along with quantitative assessments of cell viability, indicated that after 72 h incubation period, all groups of L929 cells exhibited favorable growth conditions. The group treated with 0.5 mg/ml SHI-GelMA hydrogels characterized by a relatively uniform cell distribution and a minimal presence of dead cells (**Figure 2A**). Notably, there was no statistically significant difference in cell viability between the GelMA hydrogel group and the 0.1 mg/ml SHI-GelMA hydrogel group when compared to the blank group. Research findings demonstrate that an increase of gradual enhancement in cell viability correlates with the concentration of SHI (**Figure 2B**). In experiments involving the culture of L929 cells over periods of 24, 72, and 120 hours, both the extract and conventional DMEM culture medium yielded results indicating that extracts from GelMA and SHI-GelMA hydrogels did not adversely affect the survival of L929 cells. Notably, the group treated with 0.5 mg/ml SHI-GelMA hydrogel exhibited the highest capacity for cell proliferation (**Figure 2C**). Meanwhile, phalloidin staining demonstrated that cells in the 0.5 mg/ml SHI-GelMA group exhibited enhanced spreading and a greater abundance of F-actin (**Figure 3A**).

These findings indicated that the SHI-GelMA hydrogel possesses favorable biocompatibility and that SHI exerts a beneficial influence on cell proliferation, with this effect being positively associated with SHI concentration within a defined range. Importantly, the integration of SHI with GelMA hydrogel represents an effective strategy to enhance cell loading, ensure homogeneous distribution, and promote cell proliferation. In light of these research outcomes, we have selected a GelMA hydrogel concentration of 7.5% and an SHI concentration of 0.5 mg/ml for subsequent experimental investigations.

### 3.3 *In vitro* evaluation of anti-inflammatory properties

Dysregulated inflammatory responses constitute a primary impediment to inhibit the HS. Tumor necrosis TNF-α serves as a prototypical inflammatory biomarker, whereas IL-6 plays a critical role in both inflammation and immune suppression. qRT-PCR analysis revealed that, relative to the control and GelMA groups, TNF-α and IL-6 expression levels were significantly reduced in the SHI-GelMA hydrogel group (**Figures 3B, C**). Importantly, the SHI-GelMA group demonstrated a pronounced anti-inflammatory effect.

### 3.4 Anti-angiogenic and anti-fibrotic properties

Excessive regeneration of new blood vessels, epithelial tissue, and extracellular matrix plays a significant role in the formation of HS. VEGF, a critical marker, directly regulates angiogenesis. Furthermore, α-SMA serves as a marker for the differentiation of fibroblasts into myofibroblasts, and its overexpression is linked to the development of HS.

Quantitative real-time PCR analysis revealed that VEGF gene expression in the SHI-GelMA group was significantly lower compared to the control and GelMA hydrogel groups (**Figure 3D**). Additionally, α-SMA expression was substantially decreased in the SHI-GelMA group (**Figure 3E**). These findings suggest that the SHI-GelMA hydrogel has potential efficacy in inhibiting excessive vascularization and collagen deposition.

### 3.5 *In vivo* treatment

**Figure 4:**
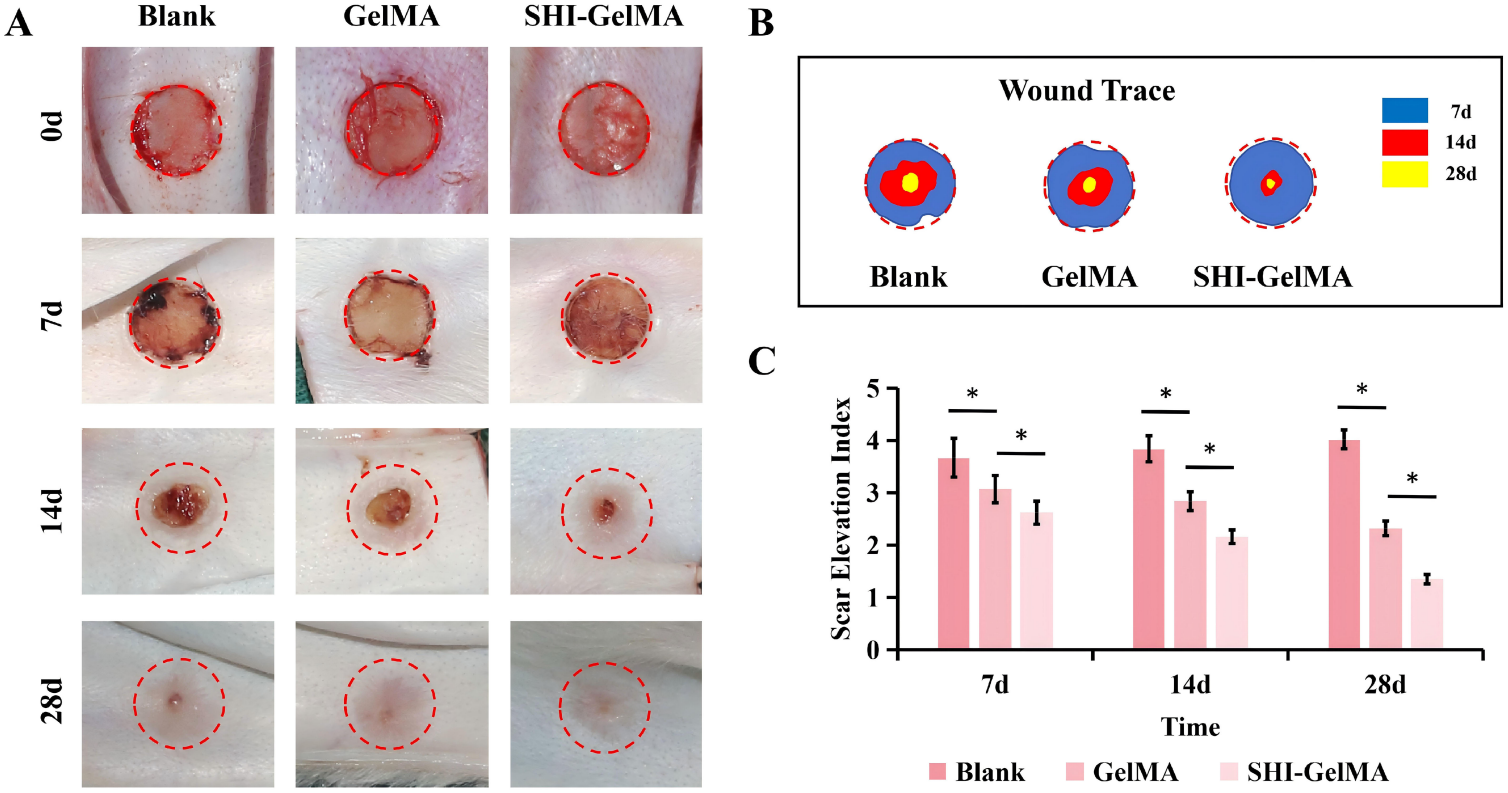
*In vivo* wound healing of SHI-GelMA hydrogel. **(A)** Gross view, **(B)** wound trance and **(C)** Scar Elevation Index treated with GelMA and SHI-GelMA hydrogel at 7,14 and 28 days. (*P<0.05).

**Figure 5:**
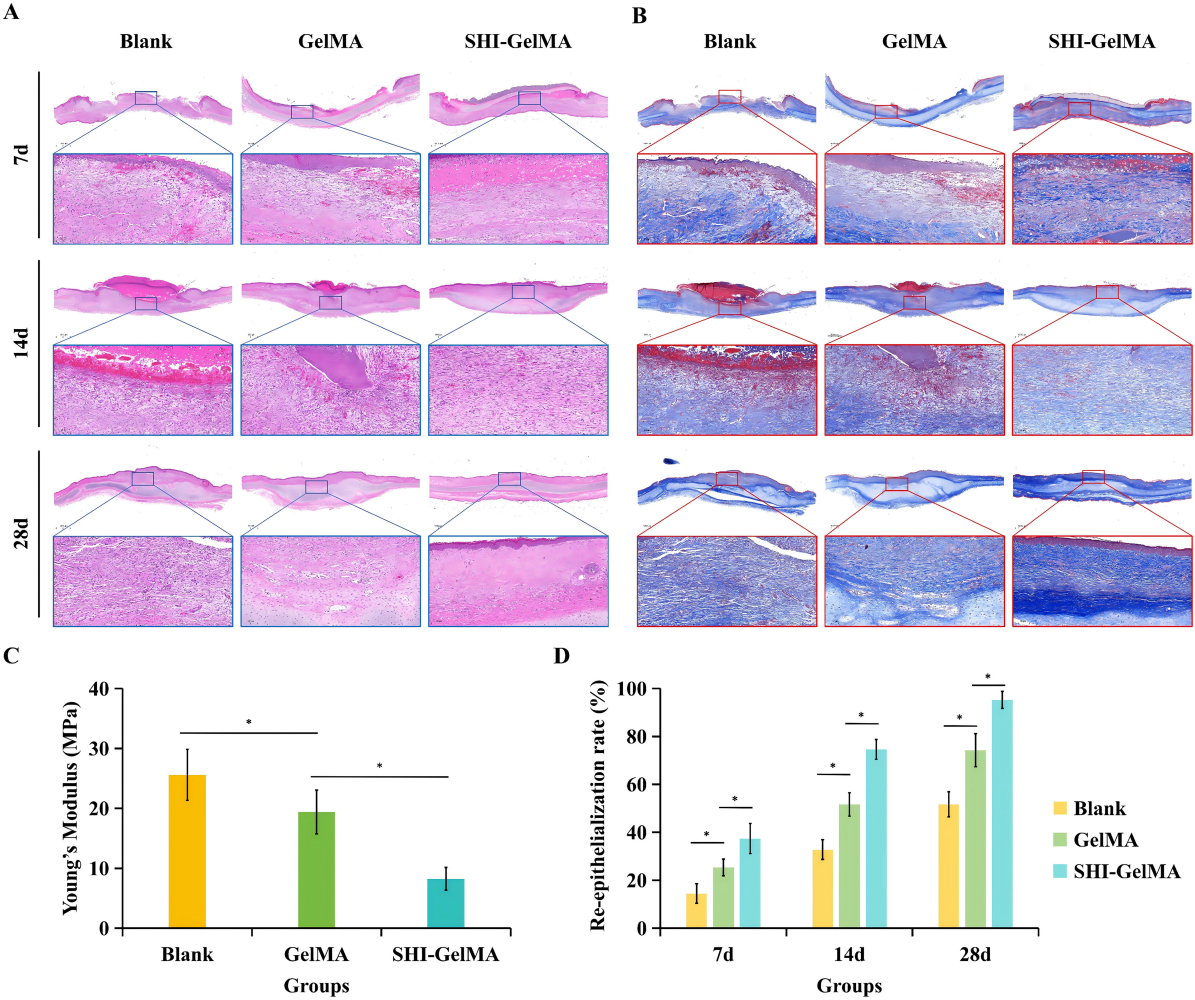
Histological staining of Blank, GelMA and SHI-GelMA groups of 7, 14 and 28 days. **(A)** HE staining. **(B)** Masson staining. **(C)** Mechanical strength of samples. **(D)** Re-epithelialization rate of samples. (*P<0.05).

We further verified through *in vivo* experiments whether the SHI-GelMA hydrogel is the optimal dressing for HS inhibition. After 7, 14, and 28 days of observation, the results indicated that the SHI-GelMA hydrogel group demonstrated significantly superior effects in promoting wound contraction and healing compared to both the blank group and the GelMA group. Analysis of the relative wound area revealed that the SHI-GelMA hydrogel group experienced approximately a 80% reduction in wound area by day 14 (**Figures 4A, B**). On the 28th day of observation, all experimental groups achieved complete wound closure. However, both the blank group and the GelMA group exhibited notable characteristics of scarring, including elevation, contraction, erythema, and palpable scars, which are indicative of HS. In contrast, the scars observed in the SHI-GelMA group were flatter, demonstrated less contraction, and exhibited reduced erythema. Furthermore, SEI for the SHI-GelMA group was quantitatively assessed at 1.35 ± 0.09, significantly lower than the SEI values of 2.32 ± 0.14 for the GelMA group and 4.02 ± 0.18 for the blank group (**Figure 4C**). Concurrently, mechanical strength testing corroborates the aforementioned findings (**Figure 5C**). These findings suggest that the SHI-GelMA hydrogel significantly reduces hypertrophic scarring and enhances regenerative healing processes.

Histological examination of wound sections was conducted using HE and Masson staining techniques to assess cellular composition, tissue architecture, and collagen deposition. The results indicated that the SHI-GelMA group exhibited a well-structured tissue organization, characterized by neatly arranged and parallel collagen fibers, along with a reduced incidence of neovascularization and inflammatory response. In contrast, the blank group and GelMA group displayed disorganized collagen bundles, excessive collagen accumulation, pronounced inflammatory responses, and significant neovascularization (**Figures 5A, B**). Based on the histological findings, we calculated the rate of re-epithelialization (**Figure 5D**).

**Figure 6:**
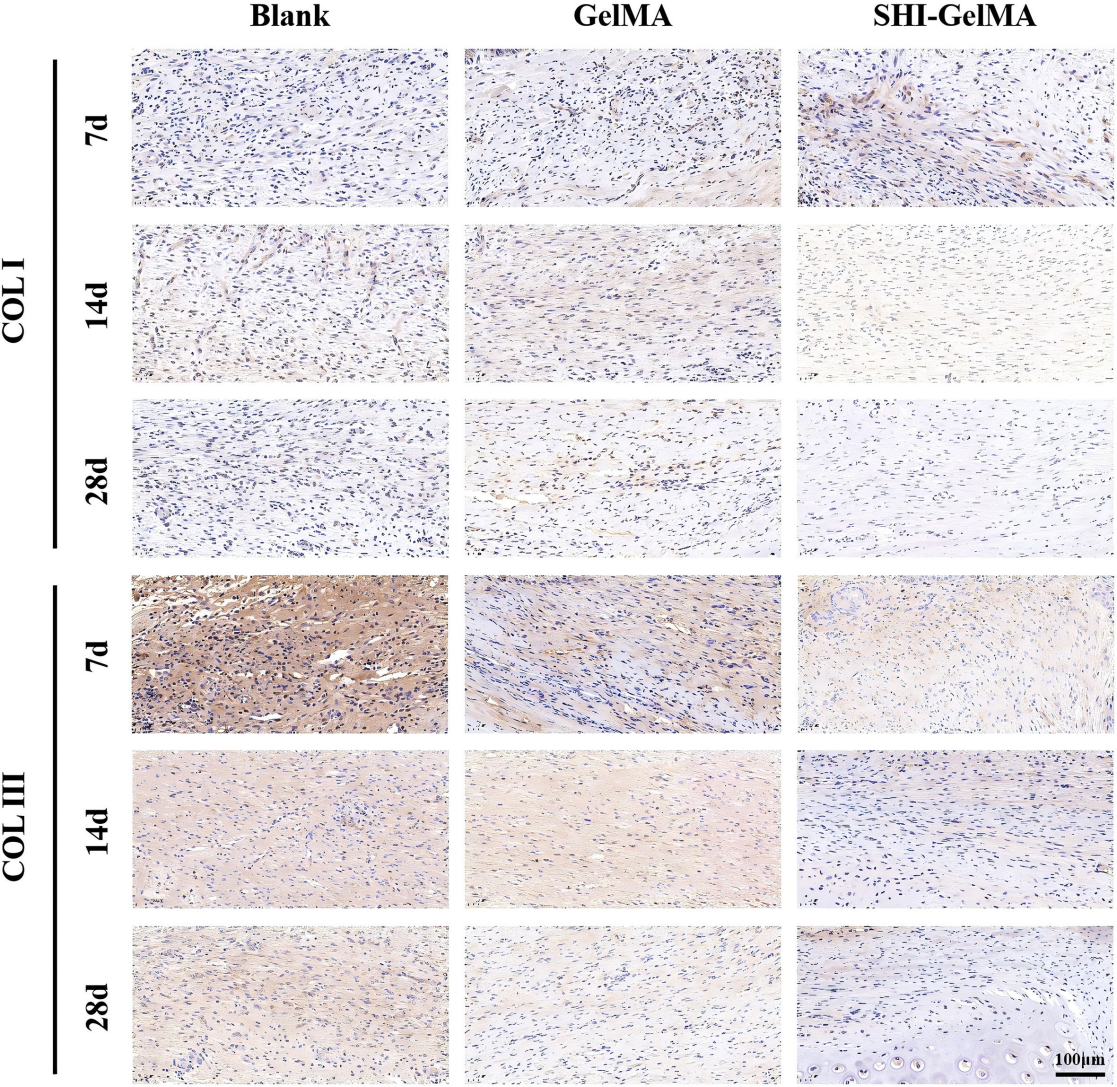
Immunohistochemistry staining of COL I, COL III of Blank, GelMA and SHI-GelMA groups of 7, 14 and 28 days.

**Figure 7:**
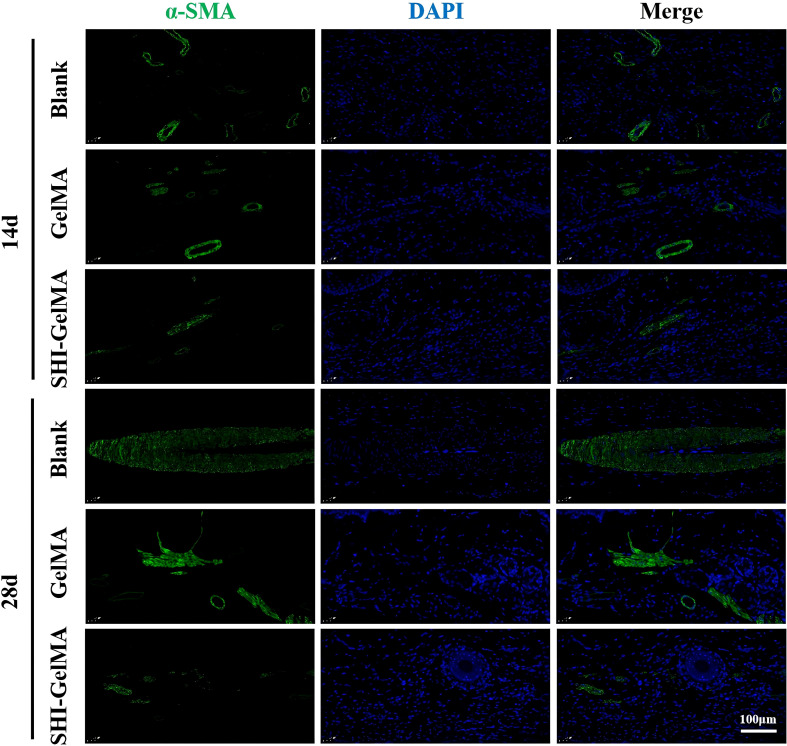
Immunohistochemistry staining of α-SMA of Blank, GelMA and SHI-GelMA groups of 14 and 28 days.

**Figure 8:**
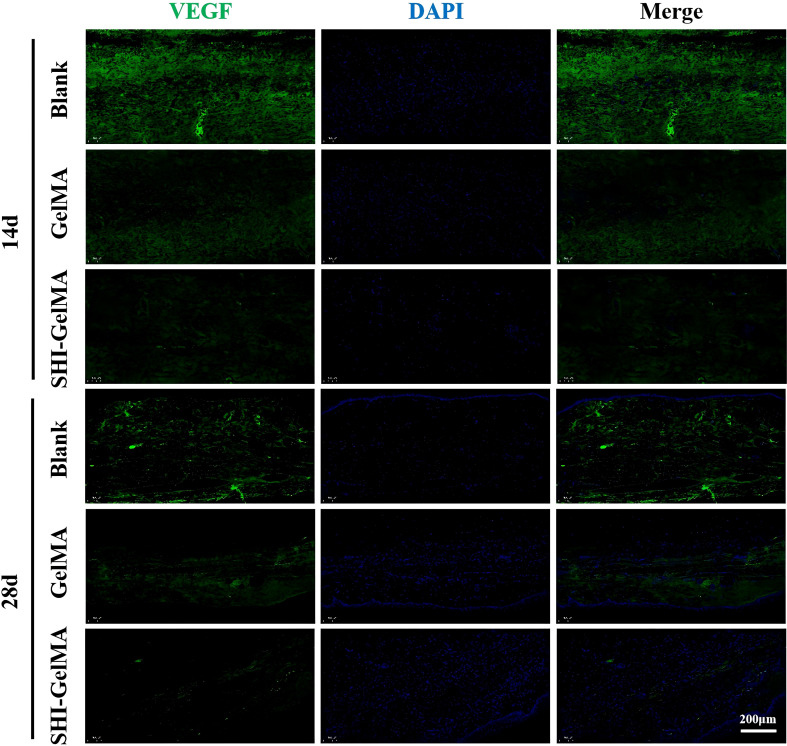
Immunofluorescence staining of VEGF of Blank, GelMA and SHI-GelMA groups of 14 and 28 days.

**Figure 9:**
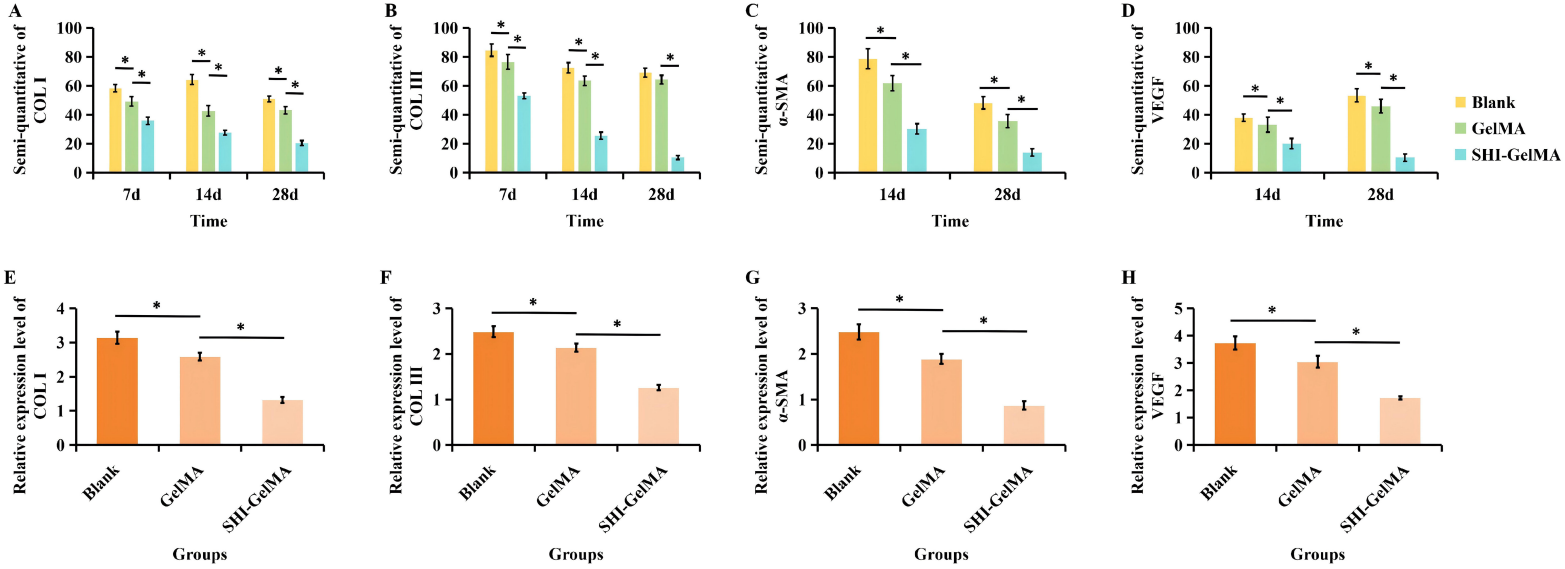
Semi-quantitative and qRT-PCR analysis of three groups samples. **(A)** Semi-quantitative of COL I. **(B)** Semi-quantitative of COL III. **(C)** Semi-quantitative of α-SMA. **(D)** Semi-quantitative of VEGF. **(E)** The expression levels of COL I. **(F)** The expression levels of COL III. **(G)** The expression levels of α-SMA. **(H)** The expression levels of VEGF. (*P<0.05).

The process of HS formation is significantly influenced by the formation of new blood vessels, epithelial tissue, and the extracellular matrix. COL I serves as the primary structural collagen in the skin, playing a crucial role in maintaining skin integrity. Meanwhile, COL III stimulates cell proliferation and tissue regeneration during the wound healing process. It provides essential structural support and nutrients to the wound, accelerates the healing process, and reduces scar formation. Furthermore, α-SMA is recognized as a marker indicative of the differentiation of fibroblasts into myofibroblasts, with its overexpression linked to the formation of proliferative scars. Meanwhile, VEGF is a biomarker of angiogenesis. The findings from the immunohistochemical analysis indicate that the expression levels of COL I and COL III in the SHI-GelMA group were significantly reduced compared to both the blank group and the GelMA group (**Figure 6**). Additionally, the expression of α-SMA in the SHI-GelMA group was found to be exceedingly low (**Figure 7**). The results of the immunofluorescence staining for VEGF demonstrated a similar pattern to that of α-SMA, with the expression level in the SHI-GelMA group being markedly lower than that observed in the blank group and the GelMA group (**Figure 8**). The corresponding semi-quantitative results are presented in **Figures 9A–D**.

QPCR analysis indicated a significant decreased in the expression of COL I and COL III in the SHI-GelMA group compared to both the blank and GelMA groups. Meanwhile, the expression of α-SMA was markedly decreased in the SHI-GelMA group. Notably, the expression of the VEGF gene was significantly lower than that observed in both the blank and GelMA hydrogel groups. In summary, the SHI-GelMA hydrogel demonstrates the potential to anti-inflammatory, anti-angiogenic, and collagen deposition-inhibiting properties (**Figures 9E-H**).

## 4 Discussion

HS frequently arise as a consequence of pathological healing, presenting a dual challenge in clinical management. On one hand, the contraction of these scars can lead to significant skin dysfunction, greatly diminishing patients’ quality of life. On the other hand, the abnormal aesthetic characteristics of HS may induce psychosocial stress, thereby exacerbating the overall burden of the condition. Current clinical interventions are limited in their ability to alleviate symptoms and may be associated with adverse effects, such as skin atrophy and loss of pigmentation (37). Furthermore, there is a lack of standardized treatment protocols. Consequently, the development of preventive treatment strategies that target the underlying pathological mechanisms of HS formation is a critical area of research within regenerative medicine. There is an urgent need to establish novel preventive and therapeutic intervention strategies. This research presents the development of an efficient wound dressing through the integration of SHI with GelMA. This combination results in a multifunctional hydrogel characterized by anti-inflammatory properties, anti-angiogenic, and facilitate collagen remodeling. The findings indicate that the SHI-GelMA hydrogel exhibits favorable biocompatibility. Furthermore, experimental outcomes reveal that SHI-GelMA can promptly and effectively modulate inflammation, actively promote collagen remodeling and anti-angiogenesis, thereby significantly reducing the risk of HS during the early stages of wound healing.

In this study, the GelMA hydrogel serves a synergistic function that extends beyond its role as a mere drug carrier. Its multifunctional properties provide an essential component of the experimental framework. GelMA facilitates the physical encapsulation and sustained release of SHI through its three-dimensional network structure. Based on *in vitro* experimental results, the concentration of GelMA was optimized to 7.5%. This is a level that balances mechanical strength and pore architecture, thereby effectively mitigating the initial burst release of the drug. Concurrently, GelMA facilitates a relatively synchronized release of the drug and degradation of the material at this concentration. Importantly, the biomimetic three-dimensional microenvironment provided by GelMA actively promotes orderly cell adhesion, migration, and proliferation, thereby offering physical support for early wound healing (38, 39). The synergistic interaction between GelMA and SHI enhances therapeutic efficacy by enabling temporal regulation that facilitates tissue repair during the early healing phase while inhibiting pathological fibrosis in the later stages. Furthermore, the mild photo-crosslinking process employed preserves the chemical stability and bioactivity of SHI to a significant extent. Consequently, GelMA functions not only as an excellent carrier for SHI but also as a foundational platform for combined prevention and treatment strategies. The integration of these two components establishes a novel and innovative basis for a preventive anti scar approach.

During the formation of HS, a significant bidirectional regulatory relationship exists between α-SMA and the inflammatory response (40). In the early inflammatory stage of scar formation, locally infiltrating inflammatory cells release various pro-inflammatory cytokines that induce fibroblasts to differentiate into myofibroblasts, thereby promoting the upregulation of α-SMA expression (41). α-SMA-positive myofibroblasts not only contribute to the excessive proliferation of scar tissue by enhancing their contractile ability and synthesizing large amounts of extracellular matrix, but they can also release inflammatory mediators through paracrine mechanisms (42). This process further recruits inflammatory cells and sustains a local chronic inflammatory microenvironment. Therefore, the abnormally high expression of α-SMA is both a consequence of the induced inflammatory response and a significant factor driving the persistence of inflammation, which exacerbates scar proliferation. In this study, we conducted immunofluorescence analysis of α-SMA in different experimental groups at 7, 14, and 28 days, leading to the following conclusions: In the blank group, the sustained high expression of α-SMA resulted in prolonged inflammation, ultimately contributing to the formation of proliferative scars. In the GelMA group, the expression of α-SMA continuously decreased, with an even more pronounced reduction observed in the SHI-GelMA group. This suggests that SHI-GelMA interfered with the activation of myofibroblasts from the early stages of inflammation, thereby affecting α-SMA expression and controlling the progression of inflammation, which in turn influences the formation of proliferative scars.

Furthermore, VEGF contributes to the excessive proliferation of microvessels, resulting in erythema and rigidity in the affected scar tissue (15, 43). Genetic predisposition, along with factors such as wound infection and delayed epithelialization, further exacerbates these pathological processes, ultimately leading to the development of raised, erythematous, and inelastic scars that are confined to the original wound margins. In the early inflammatory stage of scar formation, local inflammatory cells and fibroblasts significantly secrete VEGF in response to wound stimuli or pro-inflammatory factors (44). This secretion induces the formation of abnormal neovascularization within the dermis. These newly formed blood vessels supply essential nutrients and oxygen to the scar tissue, further promoting the excessive deposition of extracellular matrix components, such as collagen (45). Moreover, the abnormally high expression of VEGF in proliferative scars maintains elevated vascular permeability, leading to the continuous infiltration of inflammatory cells and the abnormal accumulation of growth factors. This creates a chronic inflammatory microenvironment that causes the scar tissue to thicken, become congested, and lose its normal skin structure. Studies have demonstrated that the expression level of VEGF in proliferative scar tissue is significantly higher than that in normal scars or mature skin, and its abnormal persistent secretion is closely related to the degree of vascularization of the scar (46). Therefore, VEGF is not only a crucial driving factor for neovascularization in the early stages of wound healing, but its abnormally high expression also plays a key role in the excessive proliferation and ongoing development of scars. In this investigation, we examined the alterations in VEGF levels across various groups and time intervals using immunofluorescence staining techniques. At 14 days post-wound repair, the expression levels of VEGF in the SHI-GelMA group were found to be lower than those in the other groups with the exhibiting particularly marked inhibition. By day 28, the expression pattern of VEGF remained consistent with that observed at day 14; however, the levels in the SHI-GelMA group were significantly reduced. These findings suggest that SHI substantially suppresses VEGF production during the initial phases of wound healing. SHI demonstrated a pronounced inhibitory effect on angiogenesis, effectively obstructing a critical factor in the formation of proliferative scars. This evidence provides strong support for the management of scarring during the early stages of wound healing.

The underlying mechanisms of this HS are associated with the abnormal expression of COL I and COL III. During the initial stages of wound healing, COL III, which is a thinner and more pliable form of collagen, is produced in excess and establishes a provisional matrix. This matrix is typically replaced by the thicker and more robust COL I in healthy tissue (47). However, in hypertrophic scarring, this transition is impaired: COL III remains in excess during the early healing phase, and while COL I eventually becomes predominant, its deposition manifests in a dense, parallel bundle configuration rather than the typical basket-weave pattern observed in normal skin. Moreover, elevated concentrations of COL I can stimulate the integrin signaling pathway in fibroblasts, which not only enhances collagen production but also suppresses cellular apoptosis (48). In this research, the SHI-GelMA composite hydrogel exhibited a remarkable ability to not only inhibit the excessive accumulation of COL I and COL III but also to promote the organized alignment of collagen fibers. The findings obtained from both qPCR *in vitro* and sample analyses *in vivo* provide substantial evidence to support this assertion.

This study demonstrated that SHI-GelMA simultaneously facilitates early wound healing while inhibiting late-stage scar hyperplasia. This dual effect is attributed to the composite dressing’s capacity for temporally and multi-stage precise regulation of the wound repair process. The underlying mechanism follows a coherent and logical sequence. During the early phase, the anti-inflammatory properties of SHI, combined with the moist three-dimensional microenvironment provided by GelMA, promote moderate cellular proliferation and orderly migration, thereby enabling rapid epithelialization and effective infection control. This establishes a favorable foundation for subsequent tissue repair. In the mid to late stages, the sustained release of SHI mediated by GelMA assumes a central regulatory role. Specifically, it inhibits pathological angiogenesis, thereby restricting the excessive blood supply necessary for scar hyperplasia. Concurrently, through its anti-fibrotic effects, it directly reduces excessive collagen deposition and facilitates its organized arrangement. Consequently, SHI-GelMA does not merely accelerate wound healing. It actively orchestrates an orderly repair microenvironment during the early phase and precisely suppresses fibrosis and aberrant angiogenesis during the critical later stages. This material-based, physiologically aligned temporal drug delivery and microenvironmental regulation constitute the fundamental mechanism by which this system effectively prevents HS.

Although the current research has successfully developed an SHI-GelMA hydrogel that exhibits anti-inflammatory, anti-angiogenic, and collagen deposition inhibition properties, certain limitations remain in our ongoing investigations that require further exploration and refinement. Firstly, the absence of direct spectroscopic characterization of chemical structure of SHI post-crosslinking constitutes a limitation of the present study. Nevertheless, the bioactivity data obtained provide robust indirect evidence supporting the preservation of principal functional structure of SHI. Secondly, the relatively short observation period constitutes a limitation of this study, as it precluded the evaluation of the long-term safety and stability of SHI-GelMA. Furthermore, the currently identified optimal concentration is primarily determined based on preparation feasibility and *in vitro* cellular responses, and that its therapeutic efficacy in animal models remains to be conclusively established through systematic comparisons across different concentrations.

We hereby confirm that future studies will incorporate a comparative analysis of the chemical structure of SHI before and after GelMA encapsulation and crosslinking, employing FTIR and NMR spectroscopy as essential core characterization techniques. Furthermore, a primary objective for future research is to conduct animal experiments extending for three months or longer to systematically investigate the material’s degradation process, long-term biocompatibility, and the maturation of scar tissue. Additionally, we will conduct a more comprehensive analysis of GelMA and SHI concentration.

## 5 Conclusions

The primary aim of this research is to develop an innovative preventive scar dressing that not only conforms precisely to the affected area but also exhibits anti-inflammatory, anti-angiogenic, and anti-fibrotic properties. This approach seeks to effectively reduce the development of HS during the early stages of wound healing. Experimental findings indicate that SHI-GelMA demonstrates significant efficacy in both *in vitro* and *in vivo* settings, successfully preventing the formation of HS through various mechanisms. Based on the results of this investigation, it is suggested that SHI-GelMA has the potential to overcome the limitations of conventional treatment modalities and holds considerable promise for clinical applications. Consequently, it may emerge as a highly effective solution for the prevention and inhibition of hypertrophic scar formation.

## Data Availability

The original contributions presented in the study are included in the article/**Supplementary Material**. Further inquiries can be directed to the corresponding authors.

## Supplementary material

Supplementary Figure 1 Quantification of cell survival area after L929 treated with 1.0mg/mL SHI concentrations at 3 days.
