# Supplementary figures and images for: A shikonin-based hydrogel dressing exhibiting synergistic anti-inflammatory, anti-angiogenic, and anti-fibrotic effects for the prevention of hypertrophic scars
Source: Front Immunol. 2026 Jan 14;16:1736748. doi: 10.3389/fimmu.2025.1736748 (PMC12847347; doi:10.3389/fimmu.2025.1736748)

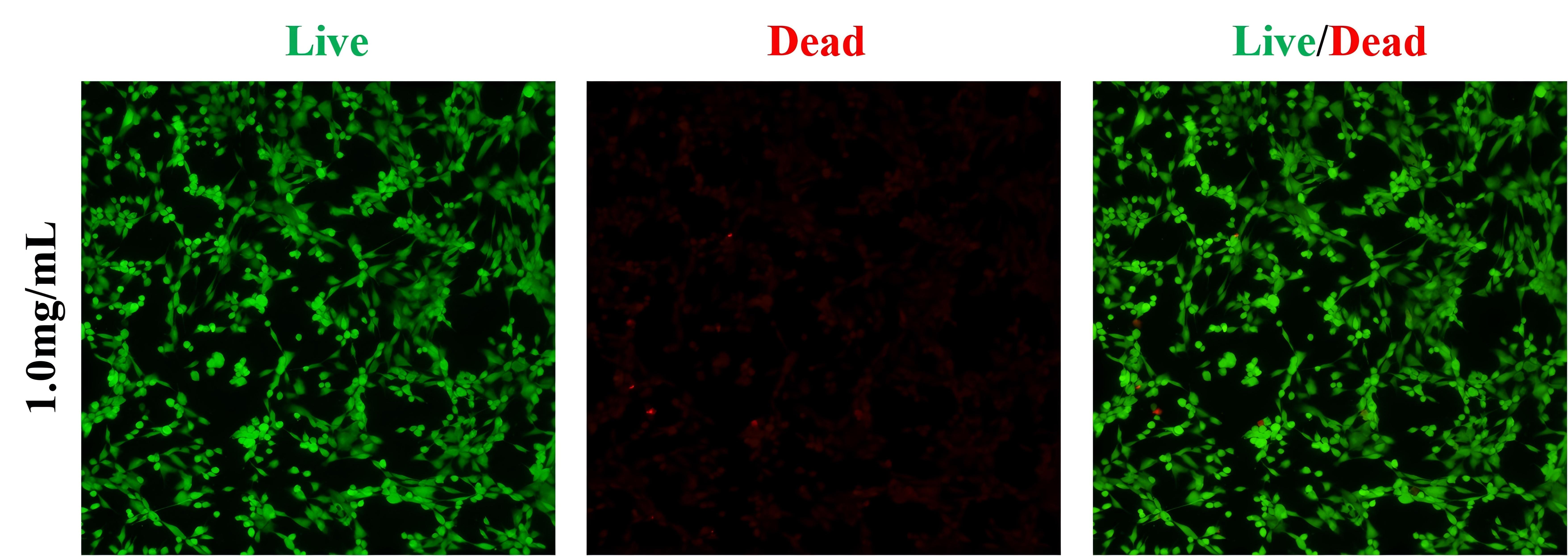

Supplement: Supplementary Figure 1 — Quantification of cell survival area after L929 treated with 1.0mg/mL SHI concentrations at 3 days. [file Image1.jpeg]
